# Unified and pluralistic ideals for data sharing and reuse in biodiversity

**DOI:** 10.1093/database/baad048

**Published:** 2023-07-18

**Authors:** Beckett Sterner, Steve Elliott, Edward E Gilbert, Nico M Franz

**Affiliations:** School of Life Sciences, Arizona State University, 427 E Tyler Mall, Tempe, AZ 85281, USA; School of Life Sciences, Arizona State University, 427 E Tyler Mall, Tempe, AZ 85281, USA; School of Life Sciences, Arizona State University, 427 E Tyler Mall, Tempe, AZ 85281, USA; School of Life Sciences, Arizona State University, 427 E Tyler Mall, Tempe, AZ 85281, USA

## Abstract

How should billions of species observations worldwide be shared and made reusable? Many biodiversity scientists assume the ideal solution is to standardize all datasets according to a single, universal classification and aggregate them into a centralized, global repository. This ideal has known practical and theoretical limitations, however, which justifies investigating alternatives. To support better community deliberation and normative evaluation, we develop a novel conceptual framework showing how different organizational models, regulative ideals and heuristic strategies are combined to form shared infrastructures supporting data reuse. The framework is anchored in a general definition of data pooling as an activity of making a taxonomically standardized body of information available for community reuse via digital infrastructure. We describe and illustrate unified and pluralistic ideals for biodiversity data pooling and show how communities may advance toward these ideals using different heuristic strategies. We present evidence for the strengths and limitations of the unification and pluralistic ideals based on systemic relationships of power, responsibility and benefit they establish among stakeholders, and we conclude the pluralistic ideal is better suited for biodiversity data.

## Introduction

Biodiversity science exemplifies the triumphs and struggles of researchers attempting to achieve ‘big data’ by combining many smaller datasets from different sources (1, 2). Millions of people, including professional scientists and non-academic enthusiasts, have collected billions of observations of biological species across the planet, many of which are available today using open databases online (3). Generally called ‘species occurrence observations’, these data document the presence of organisms of particular species at particular places and times. Many observers also record further information about the organism, such as its phenotypic traits, genetics or ecological interactions, and link this information to preserved physical samples, digital images or audio recordings (4).

Researchers, funders and policymakers aim to make biodiversity data reusable because the data contain valuable information for societal goals such as sustainable development, ecosystem services, pathogen monitoring, wildlife management and education, to name just a few (5). A prominent strategy is to create and maintain shared digital infrastructures that data collectors can use to publish their observations, e.g. by individually uploading information or contributing to institutional collections that in turn submit their records to an aggregator database. The Global Biodiversity Information Facility is perhaps the largest biodiversity data infrastructure in terms of the number of observations, with 2.3 billion records publicly available online as of May 2023.

Biodiversity science, however, continues to face several challenges to enabling data reuse, for which online accessibility of datasets is generally necessary but not sufficient (6–9). One challenge is that a wide range of actors collect biodiversity data in a decentralized manner for heterogeneous purposes, including systematists describing new species, government agencies monitoring ecological change and conservation organizations or businesses making decisions about protected species or ecosystems (10). These actors typically differ in how they locally label, preprocess and maintain their records in the context in which the data were collected and first stored (1). Moreover, the categories people use to describe what they have observed, such as species names and classifications, phenotypic traits and sampling procedures, are also frequently in flux and contested (11).

A second challenge consists of the varying institutional constraints that users place on biodiversity data. These constraints include, e.g., legal or organizational policies that restrict access due to rare species protections or protocols associated with the United States National Park Service, Indigenous or private lands. Some policies also require particular data standards and categories and impose differing norms about what makes the data fit-for-use as evidence (12). As a result, there is a substantial gap between merely storing biodiversity data records in one place and harmonizing them into a coherent and trustworthy body of information suitable for reuse across situations that vary in scale, scope, target audience or domain of application.

A third challenge underlying these first two is that scientists and other stakeholders often overlook the variety of approaches to addressing these challenges, often defaulting to one option—e.g. the ideal of having all the relevant standardized data in one place—without well-informed deliberation about the alternatives and their suitability to the social and technical context at hand (13, 14). For example, global-level projects launched in the past several decades have frequently pursued ideals of centralizing biodiversity data in a few global infrastructures under unified schemes for metadata, infrastructure management and other aspects of governance. This approach often appears effective for short-term goals of data integration and publication but creates longer-term, path-dependent constraints on science, narrowing the range of what biodiversity data stakeholders can conceive of as possible alternative strategies.

We address these challenges by providing a novel conceptual framework with which to characterize how biodiversity data infrastructures enable data reuse by adopting different sets of epistemological ideals and heuristics, and how they might be redesigned to better meet the needs and goals of practitioners. We draw on tools from the philosophy of science to develop this framework, which includes a general concept of data pooling, a continuum of organizational models for pooling and two regulative ideals that can be used to evaluate the desirability, design and effectiveness of any particular pooling model.

Our strategy to present and advocate for this framework is as follows. In the ‘Biodiversity data pooling and portals’ section, we introduce the idea of data pooling and review its importance for species occurrence observations. In the ‘Epistemic-organizational models for data pooling’ section, we describe five types of epistemic-organizational models to characterize how a diverse range of infrastructures navigate the technical, infrastructural and governance challenges of integrating data from multiple sources. We illustrate how these models differ according to their relative degree of infrastructure standardization, on the one hand, and user customization on the other. Building on these models, the ‘Regulative ideals for data pooling’ section presents two regulative ideals for biodiversity data—unified and pluralistic data pooling. In the ‘Unification for biodiversity data and its limitations’ and ‘Pluralistic data pooling as an overlooked ideal’ sections, we then examine how regulative ideals for data pooling and their corresponding epistemic-organizational models have been influential in biodiversity data pooling, and we review existing arguments addressing how effectively these ideals address challenges for making biodiversity data reusable. The pluralistic ideal we characterize provides an effective but often overlooked basis for data pooling practices and infrastructure that suit the needs of pluralistic science and societal decision-making.

## Biodiversity data pooling and portals

Biodiversity data are necessary to address long-standing questions in the life sciences and to inform policy and regulatory actions, from climate change to zoonotic diseases to biodiversity loss. The challenges created by decentralized, large-scale biodiversity data collection and sharing are as much social as technical in character, and making biodiversity data comprehensively available and reusable will likely require major changes to the cultures, organizations and infrastructures of the research communities involved (1, 14–17). In this section, we develop an account of data pooling that emphasizes the processes, practices and ideals by which people pool data into shared repositories rather than treating datasets as static objects abstracted from ongoing use and modification. We also discuss data portals as a critical type of scientific knowledge infrastructure that facilitates access to and management of pooled data resources, and we briefly stress the importance of data pooling and portals for biodiversity research and action.

We introduce the term ‘data pooling’ to describe this process by which a group of actors assemble a body of data and manage it as a shared resource for long-term reuse by others. We define ‘data pooling’ for biodiversity data as a process that combines data from multiple sources into one taxonomically standardized body of information, provides infrastructure for managing and accessing the combined data and governs it as a shared resource for a community of users and stakeholders beyond a single research project or lab. We define ‘taxonomic standardization’ as a set of processes for verifying and re-identifying a collection of species observations as needed to ensure that they are classified in a standardized way according to a single, coherent taxonomy of choice. More generally, ‘data standardization’ (also known as data harmonization) is an established term in academic and industry data science practices (18–20).

Philosophers of science have recently discussed related ideas under the topic of data integration. They illustrate its influence on the production of scientific knowledge and of assessment of societal risks and benefits, as both increasingly incorporate modeling, experiments and exploratory and hypothesis-driven approaches (21, 22). ‘Often conceived as a problem or at least a major challenge, data integration is the activity of making comparable different data types from a huge variety of potentially inconsistent sources’ (21). Among other desirable features, these datasets should be trustworthy, fit-for-use and comprehensive to the scope of problems while enabling the exclusion of irrelevant information. This rough characterization captures some common aspects of data integration practices, but it does not address key social and organizational aspects of data pooling, especially the involvement of socio-technical infrastructure, governance and work done at scales beyond particular projects or labs.

**Figure 1.**
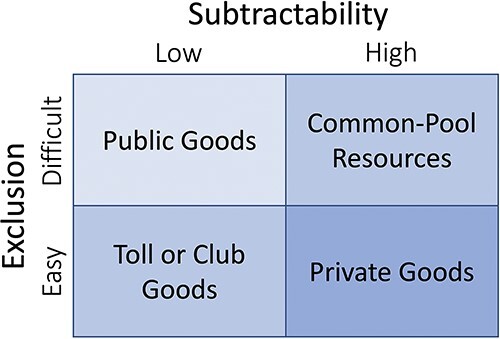
A typology for suitable management approaches to resources, modified from Ostrom *et al.* (62).

Depending on how they are managed, pooled data resources may comprise one of four types of goods under Elinor Ostrom’s classification (Figure 1). The typology is based on the degree that one person’s use of the resource subtracts from the amount of resource available to others (subtractability) and the relative difficulty of excluding people from using the resource (exclusion). Researchers generally treat data as having low subtractability because they can be copied and reused by many people without destroying or consuming the data’s information content or meaning (23). Data pooling is thus consistent with either open or restricted access to pooled resources, so pooled data can be managed as a public good or club good system, respectively. The main point of the typology is that different governance strategies will be effective depending on how the resource’s characteristics shape people’s ability to control and benefit from its use.

Our analysis explores how the standardization of resource units affects their excludability, which is critical for biodiversity science given its bottom-up organization of data collection and infrastructures. Data pooling requires infrastructure sufficient to preserve data and provide users with epistemically virtuous datasets for users’ projects. Data that are otherwise in the public domain online may nonetheless show a high degree of excludability if only a few actors are able to afford the high transaction costs needed to find them and make them useful for a particular project (24).

Data portals are the predominant mode for organizing data pooling projects with infrastructure and governance. We define ‘data portals’ as socio-technical infrastructures that include a repository, the data and metadata stored in the repository, an online Web interface for querying and retrieving stored records, an organization for administering the portal, a set of people filling roles within the organization and a set of formal and informal institutions to govern the portal, such as metadata standards, co-operative agreements with other organizations and job descriptions. People involved in running or using a portal often do many things besides running the repository, such as training new users, curating data, improving metadata classifications, hosting workshops and conducting and publishing research. Portals also have material inputs, such as funding, branding, physical facilities and sometimes preserved samples. Pooling data into a shared resource thus involves more than simply producing a new unit of information and it incorporates similar social and economic components as are found elsewhere in scientific practice.

Many countries, international organizations and scientists have prioritized data pooling efforts to monitor and understand biodiversity loss, creating big science projects on unprecedented scales in this domain. The National Ecological Observatory Network (NEON), e.g., is projected to receive >$2 billion from the US National Science Foundation (NSF) over three decades to collect long-term environmental and ecological data at ∼80 sites across the USA. The Global Biodiversity Information Facility (25) has been funded by multiple countries over several decades to aggregate digital biodiversity records from museums, national monitoring programs and citizen science initiatives into a global database that as of January 2023 contains >2.2 billion records. Conservation organizations such as NatureServe in North America use information from these projects and from US state natural heritage programs to assess threats to species and ecosystems. Their work supports non-governmental conservation efforts, legal protections under the Endangered Species Act and assessment for other relevant federal policies.

Thousands of biodiversity data portals have been created with formal organizational and governance structures, and with missions aimed at benefiting basic science research, policy or conservation decision-making and public access to scientific knowledge (26). In the USA, the NSF has channeled tens of millions of dollars into the Advancing Digitization of Biological Collections program, which supports efforts to digitize, share and augment preserved specimens in museum collections. Many of these funded projects use the Symbiota software platform (27) to host ∼80 million data records and manage contributions from thousands of registered users.

Pooling data into data portals is an essential and widespread process for efforts that often cross institutional and political boundaries. Symbiota portals in the USA, e.g., are generally multi-institutional collaborations to digitize and share preserved specimen records from different collections related to a common theme, such as arthropod parasites or Californian plants. NatureServe assesses trends in species and ecosystems that cross state and national boundaries even though most of its clients have geopolitically defined jurisdictions, e.g. the North Carolina State Heritage program. Exceptions are collection-based portals run by individual, large institutions such as the American Museum of Natural History.

## Epistemic-organizational models for data pooling

In this section, we characterize five organizational models for data pooling infrastructures. All of these models contrast with data integration activities that result in private datasets held by specific labs or collaborative projects with no or limited access for people beyond the current or future members of those groups. For example, many projects integrate datasets from different model species to study a causal mechanism in a single target system, but the data and code remain desktop-based or restricted to private or commercial access rather than openly available online through a data or code repository. Projects that submit their dataset to an independent repository, such as GenBank, DataOne or Zenodo, are contributing to an external data pooling effort but are not directly contributing to the infrastructure or governance required to operate the data pool as a shared resource.

**Figure 2.**
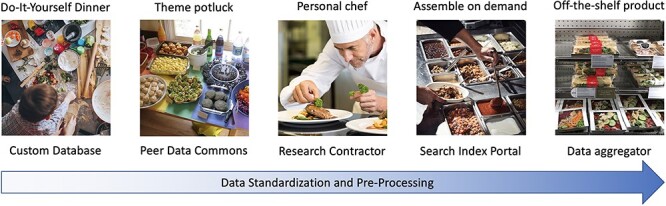
Five epistemic-organizational models for data pooling: data aggregators, search index portals, research contractors, peer data commons and custom databases. The properties of these models are illustrated by analogous approaches to how businesses or social groups make food for broader consumption. The models rely on data standardization and preprocessing to an increasing degree from left to right.

We illustrate some important differences between the models based on the degree to which they exhibit infrastructure standardization and preprocessing versus user customization and effort (Figure 2). Those features generally relate to each other inversely, so the more that a model exhibits standardization, the less it admits customization, and vice versa. However, these axes do not provide an exhaustive description of how the models are related. We review each of these models next, indicate their use in biodiversity data science, and discuss how the models involve epistemic and resource tradeoffs.

### Model 1: data aggregator

The dominant strategy for pooling biodiversity data has been to build one comprehensive infrastructure system that aims to provide a single access point and metadata system (i.e. a ‘one-stop shop’) for all data discovery and access in a domain (16, 28, 29). The aggregator model pools data from all available sources into a centralized repository. The Global Biodiversity Information Facility (GBIF) is the premiere example of an aggregator for species occurrence data, and other major examples include iDigBio in the USA and the Global Biodiversity Interaction (GloBI) database for species interaction data internationally. GBIF began in 2001 upon a recommendation from the Biodiversity Informatics Subgroup of the Organization for Economic Cooperation and Development’s Megascience Forum, and its dominant sources for occurrence data have been digital repositories for natural history collections, citizen science projects and ecological surveys hosted and conducted around the world.

This model requires substantial centralized work. In the context of biodiversity data, a centralized repository such as GBIF must handle large volumes of heterogeneous data types that are only partially standardized, often to incompatible metadata categories, and provide regular updates as data publishers contribute new observations. This operational complexity also entails an intensive design process that typically requires collaboration between many stakeholders who do not have a prior history of working together. In some cases, the obstacles prove insuperable even if stakeholders initially agree that a centralized repository is desirable, e.g. when adequate international funding and intellectual property agreements are not available to attempt a large-scale centralized database (13).

### Model 2: search index portal

An alternative to pooling all records into a centralized, comprehensive repository is to connect multiple, modular repositories under a common search interface that automates data integration based on user requests. This model can maintain the goal of comprehensive data integration while avoiding some of the major costs of the data aggregator model.

The DataONE index portal for ecological and environmental data provides a good exemplar of the search index portal model. There were originally two types of organizational members in the DataOne federated network that contributed to data pooling:

Member nodes are existing repositories of scientific data that implement services conforming to the published DataONE API… Three coordinating nodes located in the US catalog the federated content harvested from the member nodes, provide support for discovery and access, and manage replication of content among geographically dispersed member nodes (30).

The coordinating nodes are academic institutions that provide partially redundant computational infrastructure in order to ensure robust data access and preservation. Jointly, they provide the back-end system for DataONE’s core online indexing and search service through its website (dataone.org). DataONE thereby avoids having to make a single centralized repository by providing similar functionalities on top of existing domain repositories operated by other institutions, such as the NEON and Dryad Digital Repository. Users can assemble data on demand from member repositories even when the repositories rely on otherwise incompatible local software systems and heterogeneous data formats.

### Model 3: research contractor

There is a niche for research services that address the immediate needs of conservation decision-making and policy, such as deciding which parts of a landscape to prioritize for protection, documenting baseline population sizes and habitats for species affected by future development plans or justifying a change in legal protection for a species through the Endangered Species Act. These activities are subject to standards informed by government directives on the use of science in decision-making and by the possibility of having evidence challenged in court.

Conservation non-governmental organizations have developed a model to generate revenue as a contractor to pool data for others to use in research about these topics. In North America, e.g., NatureServe reported an annual revenue of $9 million in 2019–20 (31) by pooling data from clients such as US state heritage programs and public sources into a custom database and portal. As many species of conservation concern are rare or rapidly declining, research clients need high-quality standardization to avoid taxonomic identification and classification mistakes from distributed data sources. A single mistaken observation may substantially change the estimated geographic size and relevant threats for an at-risk species. Aggregators such as GBIF or DataOne do not guarantee accuracy to this granularity and instead assign responsibility for identifying and correcting errors to the authoritative data sources they use (32), which creates opportunities for custom data integration and analysis services.

### Model 4: peer data commons

While data pooling under the research contracting model is primarily driven by meeting client demands, peer data commons in biodiversity science are typically focused on increasing the supply of reusable data through research grants supporting specimen digitization or citizen science projects. The 50-plus data portals that use the Symbiota software platform (27) exemplify US peer data commons driven primarily by collections-based research funding. For brevity, we call these portals ‘Symbiota portals’ (33). They are generally led by academic researchers and are typically affiliated with consortia of museums or universities that share digitized specimen data for free public use. Symbiota portals rely on research staff and students for data contributions and management, and some use citizen scientists.

The size and scope of Symbiota portals vary widely. The Channel Islands Biodiversity Information System, e.g., covers all species collected on the islands off the West Coast of the USA, while the Symbiota Collections of Arthropods Network covers any arthropod species globally, although it primarily holds North American specimen data. Some portals are also scoped in ecological terms, such as the Great Lakes Invasive Network portal that focuses on digitized specimens of non-native plants and animals collected in North America.

Symbiota portals have key differences in governance compared to GBIF or NatureServe. The Symbiota software allows two main states for datasets within a portal: (i) ‘live-managed,’ which means that the entity owning the physical collection of specimens or vouchers has comprehensive rights within the portal to create new occurrence records and annotations, and (ii) ‘snapshots,’ which can be time-stamped versions of a portal dataset that is curated in a separate platform and can be exported to outside portals. Snapshot records can be further annotated, typically by actors who are not members of the entity that owns the physical collection. The distinction between live-managed and snapshot datasets is a matter of data governance, i.e. tracking where editing rights are vested within versus outside of a given portal.

An individual data collection typically undergoes live management in only one portal, while it may be represented as a partial or full snapshot in one or more external portals. A snapshot can be periodically (in some cases automatically) updated in external portals from its respective live-managed portal. Conversely, annotations made on snapshot occurrence records can be integrated with the corresponding live-managed collection under some social and technical conditions. For example, the Consortium of California Herbaria and the SEINet portal reciprocally exchange occurrence records and annotations. The set of Symbiota portals therefore defines a network of data pooling projects. They also have outgoing connections to other projects outside the network but no overarching aggregator devoted solely to Symbiota portal records. In contrast to organizations such as NatureServe that primarily provide services for existing data records, Symbiota portals are typically funded to digitize and curate new records to support open-ended reuse.

### Summary

The different models we have described illustrate some epistemic and organizational aspects of data pooling. One epistemic aspect involves the production of modifiable datasets, which are potential evidence for many research problems, through standardizing information from multiple sources according to a single classification system. Standardization is important when reusing data from distributed sources because distortions can arise from merging records labeled according to incompatible classifications or classifications on different levels of granularity. Some data pooling models maintain a single pooled resource, as in the aggregator case, while the search index and peer data commons models involve linking multiple pooled resources.

The organizational aspect concerns the forms of infrastructure and governance for the pooled data resource or resources. GBIF regularly resynthesizes a comprehensive taxonomy for all species in order to aggregate occurrence records from its many sources, and it does not permit individual users to modify data records in its centralized repository. Symbiota portals, by contrast, maintain a species taxonomy more directly scoped to the local data records they hold, which are typically a mix of both live and snapshot data records. Additionally, Symbiota portals rely on a shared open-source software platform, while DataONE assumes that member nodes already have their own data management and sharing software.

## Regulative ideals for data pooling

We now characterize two regulative ideals that data pooling projects may embed or presuppose. We label them the ‘unified data pooling ideal’ and the ‘pluralistic data pooling ideal’. To develop them, we draw from Grantham’s (34) account of integration as interconnectedness (while Grantham originally sought to redefine ‘unification’, his proposed meaning is closer to the current usage of ‘integration’ today). First, we review regulative ideals and how they function. Then we overview Grantham’s account of practical and theoretical interconnectedness and we discuss how those concepts relate to biodiversity data pooling. Next, we characterize general kinds of theoretical and practical interconnections within and between data pooling infrastructures and how these can be heuristically organized to achieve integration. Finally, we characterize the two regulative ideals. The following section then analyzes the suitability of these heuristics and ideals for biodiversity data pooling.

### Regulative ideals in scientific practice

A ‘regulative ideal’ is a property realized by a system or body of knowledge in its ultimate (or distant future) state of development. A regulative ideal differs from a project objective or organizational goal, which both connote achievability in relatively short timespans. Instead, the desired property is ideal because it does not currently hold nor do we expect it to in any appreciable time. The ideal property is also regulative because it spurs researchers to regularly reexamine and assess their current knowledge and practices so as to attempt to improve it. In doing so, researchers compare the features of their current body of knowledge and activities to the posited ideal feature, note the differences and work to revise the former to more closely approximate the latter. They may also alter or replace the ideal if they choose. As the ideal cannot be straightforwardly satisfied on typical time scales used for planning research projects or institutional initiatives, it spurs ongoing methodological work to develop and revise heuristic maxims to guide individual and collective efforts. While some might class regulative ideals as norms, for our purposes it is more accurate to say that they provide higher-level motivations for adopting and enforcing norms.

Most discussions of regulative ideals have at least two limitations. First, they prominently treat unification as the regulative ideal of knowledge. (The concept of regulative ideals is usually associated with Immanuel Kant, who proposed that we use our cognitive faculty of reason regulatively to examine the concepts and regularities developed in natural science and how well they fit into a single unified system of knowledge, which he took to be the ultimate ideal of all our cognition. We cannot know beforehand that such an ideal is actually achievable, and it functions to spur ever further inquiries and structuring of their results.) While there are weighty criticisms of unificationism, few have proposed alternatives that are explicitly developed as positive ‘regulative’ ideals and have clear heuristics. Philosophers often talk past each other about how well unificationist ideals describe current knowledge and should inform research design and evaluation in the short versus long terms (35–37). As a result, it can appear as if the concepts of regulative ideal and unification are necessarily conjoined, and many researchers assume this by default. Second, most discussions of regulative ideals focus on knowledge encapsulated in scientific theories composed of laws of nature for the purpose of causal explanation. For instance, researchers pursuing unification might aim to develop a knowledge system according to which they can reduce all psychological laws and concepts to biological laws, and both to those of physics.

We confront both of these limitations when developing and evaluating regulative ideals for data pooling. First, unification ideals enjoy widespread and often implicit adoption for biodiversity data infrastructures, but those ideals can stifle or obscure legitimate scientific disagreements. For example, standardizing the production and labeling of datasets often causes controversies about the extent to which whole research communities (and non-academic stakeholders) must converge on shared ontological beliefs, methodological standards, and aims (1, 38, 39). Second, much of the relevant theoretical knowledge for biodiversity data is structured in taxonomies, not in statements of causal laws or mechanisms. For data pooling infrastructures, these taxonomic theories are used primarily for structuring, ordering and searching through knowledge (40). While data users may deploy the results in further, explanatory projects, the benefits of unification for explanation versus data pooling are separate questions. Premature data unification could in fact slow down theoretical progress by erasing or distorting important differences in the properties of phenomena.

### Data pooling and interconnectedness

In order to think about regulative ideals for data pooling without presupposing views about theoretical unification, we draw on a lesser known but especially relevant account of integration by Grantham (34). In particular, Grantham characterizes how a range of scientific activities beyond intertheory reduction can advance both theoretical and practical integration. He writes that fields can be theoretically integrated as theories developed within each field become more densely interconnected and that fields can be practically integrated ‘insofar as one field comes to rely on the methods, heuristics, or data of a neighboring field’ (34). On his view, researchers can increase theory integration with new explanatory, ontological or conceptual connections such as explanatory reduction, part-whole or causal relationships, or conceptual refinements. Similarly, they can practically integrate fields by establishing dependencies among activities and resources across fields, such as by using theories or methods from one field to generate new hypotheses in another or methodological integration that uses data from multiple fields to test hypotheses. Grantham emphasizes that integration is a matter of degree based on the interconnectedness among fields.

In our case, the primary aim of data pooling is to facilitate data reuse, which is an instance of practical interconnectedness. A data pooling project provides a set of scientifically coherent data records for researchers to access from multiple different contexts, such as labs, projects, disciplines or countries. The pooling project is an authoritative source in virtue of having harmonized and augmented the information contained in the pooled data records and making these altered versions available for reuse. ‘Authoritative’ minimally means to author a dataset with distinctive content or features. More strongly, the authoritativeness of a data pool is the degree to which it is credentialed by experts contributing to the data pooling process, the methods they use and explicit validation of the data’s epistemic fitness-for-use (42).

For theoretical interconnectedness, efforts to harmonize data within and among categories can establish more coherent and principled systems of categories, e.g. by leading researchers to agree on common principles for applying Linnaean ranks to taxa. Classificatory theories express general claims about the existence and properties of many entities or processes in the world (1). They also express claims about relevant or necessary information scientists should provide in measuring or manipulating their objects of study. If researchers reach a global consensus about what there is to observe and how to observe it, then they have achieved a strong unification of the concepts, methods and beliefs involved in making and using the data.

Data pooling can also increase practical interconnectedness in a way not previously noted. When they pool data, researchers create and often maintain a new epistemic resource that the larger research community can recognize as authoritative. To the extent that researchers add or revise information for existing records (4), a pooled dataset can collect the observational and analytical outputs of many fields within a single resource. This can support but is not the same as using data from different fields to make theoretical inferences or test models (21, 22). Crucially, we use Grantham’s notion of practical interconnectedness to show that unification is not aptly contrasted with local or problem-specific integration if this entails fragmentation of the system into unconnected data silos.

### Heuristics for increased data interconnectedness

We can now discuss heuristics people use to inform how they design and manage their current data pools relative to the aim of increasing interconnectedness. Heuristics are rules of conduct for reasoning that are relatively easy to use and often pragmatically useful, but which rely on substantive simplifications or idealizations about the world, and therefore can yield systematically bad outputs despite correct application (43). We address how these heuristics relate to unified and pluralistic ideals for data pooling in the following subsection.

**Figure 3.**
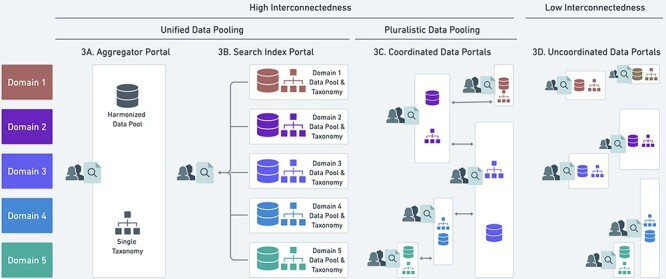
Four possible heuristics (A–D) for data pooling and their relationship to the unified and pluralistic ideals. See main text for discussion of A-D. White boxes represent distinct data portals, and colored symbols within the box represent data standardized to a local, potentially unique taxonomic classification that is not deliberately harmonized with other taxonomies. The vertical overlap in white boxes indicates where portals overlap in the scope of data they include.

Figure 3 shows the practical and theoretical interconnectedness of pooled data based on the organizational models listed in the ‘Epistemic-organizational models for data pooling’ section and their capacities to share data. The vertical axis shows how data pooling can be organized according to domains, which we define as ontological or theoretical categories. The domains scientists use to organize biodiversity data are typically taxonomic groups, such as arthropods, fungi, mammals or plants, but can include geographic regions and ecological relationships.

The horizontal access of Figure 3 shows three heuristics that achieve high interconnectedness: 3A and 3B pursue unified data pooling by implementing a single, centralized aggregator (Figure 2, model 1) or a search index portal (model 2); 3C pursues pluralistic data pooling by implementing a coordinated network of data portals consisting of one or multiple organizational models. By contrast, low interconnectedness arises in 3D when multiple data portals have a poor capacity to share data despite overlapping contents according to the domains shown.

### Relation of heuristics to regulative ideals

For data pooling, practical interconnectedness raises issues about how to organize and conduct the distribution and regulation of authoritative status on data records. Recall that regulative ideals provide shared long-term ideals against which researchers evaluate current practices and resources, so the positions below characterize bodies of knowledge at some future ideal state. A major point of contention in biodiversity data science is whether the community should have more than one authoritative source for the same data records (28, 32, 44). When researchers advocate for different answers to that question, they invoke different, frequently tacit, regulative ideals.

Unified data pooling: every repository

- has a distinct, non-overlapping set of records,
- is the authoritative source for all the records it holds,
- follows the same overarching taxonomy for classifying its records and
- defines its scope of domain according to a category in that taxonomy.

Note that condition (iii) describes how data records are classified within repositories, while condition (iv) describes how data records are grouped among repositories. For example, consider a group of museums that each host their own data portals for their specimen collections. The museums could satisfy condition (iii) by all labeling their specimens using the same taxonomy while failing condition (iv) if their collection holdings do not map cleanly onto non-overlapping taxonomic categories. Unified data pooling can be pursued through two of the heuristics discussed earlier: a single, centralized data aggregator (Figure 3A) or multiple modular repositories connected by a search index portal (Figure 3B). There is an equivalent definition from the perspective of the data records: every data record has only one authoritative repository, is standardized according to the same overarching taxonomy and is assigned to a repository according to how it is classified in that taxonomy.

An alternative, pluralistic ideal shows how moderate to high degrees of interconnectedness are possible given persistent theoretical and practical disagreements. Philosophers have noted that scientists often invoke regulative ideals other than unification, especially in explanatory contexts, and that ideals of integration are localized to particular research problems (37, 38). What might an ideal for data pooling without unification look like, especially given that data pooling does not fit philosophical accounts of explanatory unification or integration to address a local problem? To address the overarching goal of data sharing and reuse, the pluralistic ideal must characterize at least a moderate level of interconnectedness. To avoid collapsing into the unification ideal, however, it must not be a transient intermediate on the way to total unification.

Pluralistic data pooling: for any given repository,

1. it can hold overlapping data records with other repositories,
2. it is the authoritative source for a unique set of records, even when those records are shared in other repositories,
3. it can harmonize its records according to different taxonomies and
4. it can define the scope of its contents according to different domain categories than other repositories.

Pluralistic data pooling can be pursued through multiple coordinated data pools, as shown in Figure 3C, where the need for data sharing is based on overlaps between the repositories’ scopes. Any given data record can be accessed through multiple repositories, standardized according to different taxonomies and assigned to repositories according to different taxonomies. By allowing multiple access points for the same data records, researchers do not necessarily engender low theoretical or practical interconnectedness.

Pluralistic data pooling can foster fragmentation, as represented in Figure 3D, with potentially undesirable results. For example, repositories might maintain multiple versions of the same data record with no way to share updates. It might also be difficult to translate data standardized using one taxonomy into a different taxonomy preferred by another repository. Moreover, the contents of repositories may be defined such that they do not match the needs of users, increasing the burdens users experience trying to find all and only the relevant records for their purposes. Many data pooling projects also struggle to navigate the common boom-bust cycles of funding in the absence of mutual networks of support (45), and this can often be addressed by eliminating redundancies and sharing personnel or technological resources. While conceptually defensible, philosophical arguments for radical eliminativism about biodiversity (46) fail to address these practical challenges for maintaining multiple disconnected knowledge resources. As a result, biodiversity scientists generally discourage the fragmented heuristic shown in Figure 3D for open data, although it might be justified for more competitive or confidential settings.

By contrast, Figure 3C illustrates how pluralistic data pooling can approximate the positive regulative ideal through coordination among heterogenous data pooling models. In general, the coordinated approach relies on organizational and infrastructural elements that address the shortcomings of fragmentation. For data repositories that maintain authority on the same records, their managers must negotiate mechanisms for sharing updates or corrections, and the whole community must align alternative taxonomies used by repositories to ensure that data can be translated (or ‘cross-walked’) across taxonomies without loss of precision and accuracy. The community must also help set the boundaries of the repositories to ensure that data pooling projects are appropriately scoped to the aims of both data contributors and users.

Those who adopt the pluralistic data pooling ideal seek the benefits of customizing data pooling efforts in concert with tolerance for the preferred scopes and taxonomies of different communities. They do not enforce uniform standardization when it poorly reflects their divergent local research aims and situations. Similarly, they work to avoid the downsides of fragmentation by investing in the social and technical infrastructure needed to coordinate information across pooling projects. Insofar as the underlying heterogeneity persists in the communities using and sustaining these data repositories, realizing the pluralistic ideal achieves data integration without unification: high interconnectedness that does not lead to unified data pooling or theoretical unification.

## Unification for biodiversity data and its limitations

By documenting when and why the heuristics described in Figure 3 systematically fail, we gain a better understanding of the suitability of regulative ideals for situated scientific practices such as data pooling. Most approaches to data pooling articulated by biodiversity researchers over the past several decades pursue the centralized or modular forms of unified data pooling (28, 47–51). In this section, we illustrate both centralized and modular forms using high-profile examples involving past and present biodiversity data (28, 52). We analyze some heuristic guides for collective action that accompany these ideals and present several arguments that these assumptions are not met for biodiversity data.

**Figure 4.**
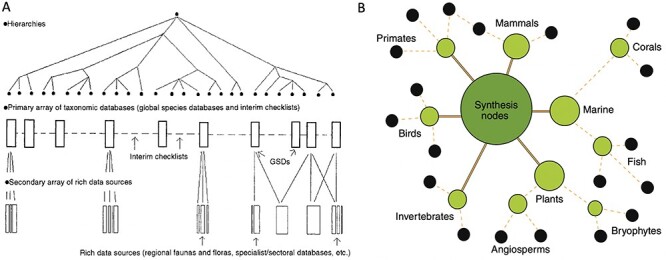
Two heuristic approaches to unified data pooling. (**A**) Image from Bisby (28), illustrating his envisioned unified hierarchy of classificatory theories for taxonomic domains. The hierarchy would enable a centralized aggregator or modular system of data pools. (**B**) Image from Gallagher *et al.* (52), illustrating a modular hub-and-spoke system for integrating trait data about species by taxonomic domains. Black circles at the edges of the network show primary data publishers or sources. Intermediate nodes show projects harmonizing data and developing standards. The central, largest node represents a trait dataset registry that functions as a basic search index portal across domains.

We first consider a particular heuristic for unified data pooling articulated by an early leader in biodiversity informatics, Frank Bisby, who aimed for a comprehensive online ‘catalog of life’ for all species (28). Bisby argued that a universal taxonomic hierarchy (Figure 4A) was essential to pooling biodiversity data on a global scale given the institutionally and geographically distributed sources of species observations. In particular, he advocated against maintaining multiple overlapping classifications for species, e.g. regional flora or fauna lists of species produced by individual experts or local groups (28). The assumption was that globally distributed data records would then be produced and updated according to this universal classification system. GBIF continues Bisby’s work to construct a comprehensive taxonomic ‘backbone’ for all species. Making this vision a practical reality has been a critical challenge for GBIF’s goal of pooling species observations in a centralized repository for public access.

More recently, Gallagher *et al.* (51) advocate for a unified ideal while rejecting the goal of constructing a single, comprehensive repository. They introduce the Open Traits Network (OTN) as an international collaboration focused on data synthesis for organismal trait information. They contrast their approach against that of a global aggregator:

A centralized and connected network structure will not facilitate trait data synthesis. Trait observations are highly nuanced and hierarchical. Describing multiple aspects of a phenotype for any organism with traits is not amenable to a simplified set of exchange fields that apply across the Tree of Life (51).

In short, and in contrast to species observations as organized in GBIF, trait observations from different taxonomic groups are too heterogeneous to pool into one database under a single standardized classification system.

OTN’s strategy is closer to DataONE’s approach to linking ecosystem, climate and species data, but with a stronger emphasis on data pooling according to a progressive hierarchy of domains (Figure 4B). While Gallagher *et al.* (52) do not directly address the issue of pluralistic authorship for the same trait observation records, they endorse the goal of all data being curated according to a universal set of categories: one of OTN’s five major aims is to ‘build consensus for common trait definitions and measurement methodologies for major organismal groups’ (52).

Given these examples of influential teams adopting the unified data pooling ideal, it is worth considering how their heuristic strategies may systematically fail and lead to substantial practical and epistemic costs. A core component of unified data pooling is that ideal data pools will have a consensus classification scheme for all relevant data and repositories. Scientists typically argue for the ultimate reality of a consensus scheme by appealing to universal truths about the nature of the domain, e.g. the existence of a tree of life, or to the characteristics of reliable knowledge in the domain, e.g. a structural versus evolutionary approach to defining anatomical features (39). The implication is then that everyone will benefit from unified pooling data because the resulting dataset can be constructed based on an objective, universal way of ‘carving up’ nature. When researchers adopt heuristics to approximate the unified data pooling ideal, they reason that since the ideal pools will share a consensus classification, they can approximate this ideal state by implementing consensus classifications in the design and management of current and future data pools.

We briefly review a few existing arguments for why this line of reasoning has proven problematic for biodiversity data (10, 32, 53–57, 58 and references therein). First, it assumes that researchers have a consensus classification now or that they will sometime soon, neither of which has come true after centuries of research in systematics. Furthermore, a consensus classification presumes that the categories are sufficiently stable over time and not subject to major disputes (59), which is not the case even for highly studied groups such as birds or mammals (11, 54, 55).

Second, there is an assumption that the consensus classification provides a set of domains for data pooling that are equally suitable for all problems, or more realistically the problems the research community deems important enough to prioritize. Recognizing that not all research problems are treated equally in practice raises questions about who decides which ones are important, raising concerns about equity, justice and sovereignty given that the currently prioritized problems, aims and researchers pursuing them are not representative of society at large (56). Third, there is a potential mismatch between the domain categories most preferred by data users versus collectors and curators (32). Fourth, unified data pooling presumes that the useful information contained in datasets sourced from heterogeneous contexts can be preserved during the standardization process (32).

In addition, while unified data pooling has strengthened collective efforts to digitize and improve data quality, it also entrenches a particular epistemic-organizational division of labor and responsibility that can be difficult to alter and that privileges the classificatory decisions of those with the most institutional power (10, 32). Centralized biodiversity portals such as GBIF and GenBank typically restrict curation privileges for users and either outsource editing of records to other institutions or allow participants to edit a subset of data (e.g. only the data people have contributed themselves). These restrictions impact the accuracy of data across scales and its tunability for different applications (53, 60). Restrictions on editing centralized datasets can arise for a variety of reasons, including constraints from original data sources on modifying their content or the difficulty of vetting expert users on that scale. Users with corrections or new annotations must then contact each original data source individually to request edits.

Finally, few biodiversity experts work on all groups and at the global level. Instead, experts and communities tend to have taxonomic, geographic and governmental boundaries more accurately represented at low- or middle-level scales. Research communities that do analyze all organisms at the global level usually lack the corresponding situated expertise needed, e.g., to reconcile non-congruent classification schemes inherent in biodiversity data packages aggregated from multiple localized sources and communities of practice. The result is a primarily one-way flow of new and edited data from many distributed sources toward the global dataset with limited gain for particular communities to collaborate on data curation and to preserve improvements across individual projects. The community that focuses on ‘global data’ is small proportional to the whole field, and just because it claims wide epistemic scope, it does not thereby represent wide social–organizational scope.

## Pluralistic data pooling as an overlooked ideal

**Figure 5.**
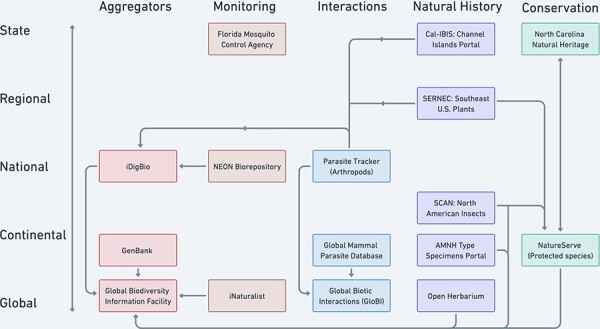
Selected examples of North American and global biodiversity data pooling projects illustrate the current partially coordinated flow of biodiversity data (uni- or bi-directional arrows). Projects are distinguished by the geographic scale (*y*-axis) and type of domain used to characterize their scope (colors by column on the *x*-axis). The aggregator column represents some important biodiversity data pooling projects whose scopes cut across the other domain categories.

In this section, we illustrate the heuristic of coordinated pluralistic data pooling in the context of biodiversity data. To begin, Figure 5 illustrates how selected current biodiversity data portals define their domains and interact through data sharing. Figure 5 describes how things are rather than directly evaluating them with respect to a regulative ideal. However, we have selected particular data pooling projects to highlight some points of divergence between unified and pluralistic data pooling. In particular, the repositories shown do not define their scopes according to a single system of non-overlapping domains, instead drawing on a variety of cross-cutting disciplinary categories such as taxonomic groups, geopolitical regions, conservation status and ecological interactions. Figure 5 also illustrates how the broader network of biodiversity data infrastructures shares and maintains multiple authoritative sources for the same observations.

Rather than eliminating the pluralistic domain classification schemes and data authorities represented in Figure 5, the pluralistic data pooling ideal treats these as virtues. The pluralistic ideal characterizes a system in which pooled datasets are customizable to the aims of specific communities and their research problems, based on infrastructures that enable researchers to laterally exchange data while maintaining different local classifications.

Symbiota portals provide an example of a network that increasingly pursues the pluralistic ideal. Each portal publishes its data on the Web using the Darwin Core standard for taxonomic categories. Any other portal can then construct a customized dataset by importing partial or full snapshots from any source, not limited to Symbiota portals, that makes data available online in a Darwin Core–compliant format. This importing process facilitates custom mapping of records for which datasets differ in their metadata categories or portal data managers can locally edit snapshot data.

Symbiota portals are developing the capacity to overcome systemic limitations to unified data pooling described in the previous section. First, each portal maintains autonomy to select and curate pooled data according to metadata information tuned to its aims and classificatory theories. No universal taxonomic classification or other classificatory theory is necessary, as data harmonization occurs only between a targeted, often small number of sources. Next, Symbiota portals can curate data locally. Compared to global portals, larger percentages of Symbiota portal users can contribute to the governance of metadata standards in the primary portal they use and maintain versions of cleaned data they produce. As a result, and third, it is possible for portal users to maintain accurate data at the level of individual species and for biodiversity monitoring projects.

To increasingly approximate the pluralistic ideal using the coordinative heuristic, at least two conditions must be met. First, the producers of classificatory theories for occurrence data must be empowered and incentivized to provide sufficient information to enable translation across alternative theories (38, 54, 61). The flexibility to customize pooled data across competing or historical viewpoints depends on implementing the capacity and professional incentives to accurately map data records annotated under one system to the categories posited by another system. In principle, this flexibility is consistent with constructing a comprehensive classificatory theory so long as the ability and resources to crosswalk data to other (perhaps less comprehensive) theories are maintained.

Second, data pooling projects must closely align with professional and enthusiast communities that have the expertise and resources to curate the resulting datasets. From an institutional perspective, enabling each community to steward its data and lead data management is a powerful incentive that is potentially lost when unified data pooling is imposed in a top-down fashion. Furthermore, a reticulated strategy for data pooling can deliver benefits over the unified approach when experts need to represent conflict and ambiguity inherent in individual sources and versions or when they disagree about how to characterize data but nonetheless wish to share data across projects. Nonetheless, maintaining standardized datasets and infrastructures is highly resource intensive, so it is generally infeasible for each person to run their own data pooling project individually.

## Conclusion

We have developed a conceptual framework with which to characterize biodiversity data pooling, the regulative ideals guiding current and past data pooling efforts and their suitability of their heuristic strategies to existing knowledge, technology and interests of salient communities. Our framework includes a general definition of data pooling, a continuum of organizational models for pooling data and two regulative ideals that inform and evaluate heuristic approaches to designing and connecting scientific data infrastructures and projects. We contrasted the strengths and limitations of each ideal for pooling biodiversity data to serve a plurality of aims. When we consider regulative ideals, we should recall that data pooling generally (almost by definition) serves a plurality of aims. Infrastructure and governance for data reuse must balance aspirations for scale, efficiency, relevance and fitness-for-use in a wide range of problems (22).

While the unified data pool has operated widely throughout biodiversity data science, approximating a global and comprehensive scale of pooled biodiversity data does not eliminate the demand for datasets scaled and attuned to local problem and solution frameworks that scientists and decision makers have to address. Nonetheless, as the coordinated network of data portals exemplifies, data can be usefully integrated without satisfying a requirement for theoretical unification. There is therefore substantial opportunity for biodiversity data scientists to overcome key limitations of existing heuristics for unified data pooling by pursuing a coordinated approach to pluralistic data pooling.

Nonetheless, all the heuristics we discussed make substantive assumptions about the social and technical states of the larger system. The coordinative heuristic assumes sufficient expertise and personnel to map concepts across taxonomies in pluralistic approaches, while the centralized aggregator and modular hierarchy heuristics rely on robust agreement about a shared taxonomy that adequately serves all community aims. This illustrates how excludability is not an inherent property of a pooled data resource but instead depends on further social and technical attributes of the system as a whole. Pursuing the unification ideal may in fact raise excludability and lower equality of access and benefits if it is implemented heuristically in ways that run systematically counter to the aims and needs of many local communities.
